# Bladder ultrasound: evidence of content validity of a checklist for training nurses

**DOI:** 10.1590/0034-7167-2023-0183

**Published:** 2024-12-16

**Authors:** Filipe Utuari de Andrade Coelho, Sabrina Martins Reigota, Flávia Manfredi Cavalcanti, Dejanira Aparecida Regagnin, Beatriz Murata Murakami, Vinícius Batista Santos

**Affiliations:** I Universidade de São Paulo. São Paulo, São Paulo, Brazil; II Hospital Israelita Albert Einstein. São Paulo, São Paulo, Brazil; III Hospital DF Star. Brasília, Distrito Federal, Brazil; IV Universidade Federal de São Paulo. São Paulo, São Paulo, Brazil

**Keywords:** Ultrasound, Validation Studies, Urinary Bladder, Nursing, Advanced Practice., Ultrasonido, Estudio de Validación, Vejiga Urinaria, Enfermería, Enfermería de Práctica Avanzada.

## Abstract

**Objectives:**

to develop and analyze evidence of content validity of a checklist for training nurses in measuring bladder volume through ultrasound.

**Methods:**

a methodological study, consisting of three stages: literature review; instrument item preparation; and analysis of evidence of content validity. The Content Validity Index (CVI) and Gwet’s AC2 were used for content validity analyses.

**Results:**

the checklist consisted of 23 items. The CVIs for clarity, relevance and dimensionality were 0.99, 0.99 and 0.98 respectively, and the CVIs for Gwet’s AC2 with coefficients for clarity, relevance and dimensionality were 0.89, 0.97 and 0.95, respectively, with p<0.001.

**Conclusions:**

the checklist developed for training nurses in measuring bladder volume through ultrasound achieved adequate evidence of content validity, and can be used to train nurses in clinical practice and future research.

## RESUMEN

**Objetivos:**

desarrollar y analizar evidencia de validez de contenido de una lista de verificación para la capacitación de enfermeros en la medición del volumen vesical mediante ultrasonido.

**Métodos:**

estudio metodológico, que consta de tres etapas: revisión de la literatura; preparación de artículos de instrumentos; y análisis de evidencia de validez de contenido. Para los análisis de validez de contenido se utilizaron el Índice de Validez de Contenido (CVI) y el AC2 de Gwet.

**Resultados:**

la lista de verificación estuvo compuesta por 23 ítems. Los IVC de claridad, relevancia y dimensionalidad fueron 0,99, 0,99 y 0,98 respectivamente, y los IVC para el AC2 de Gwet con coeficientes de claridad, relevancia y dimensionalidad fueron 0,89, 0,97 y 0,95, respectivamente, con p<0,001.

**Conclusiones:**

la lista de verificación desarrollada para capacitar a los enfermeros en la medición del volumen vesical mediante ultrasonido logró evidencia adecuada de validez de contenido, y puede ser utilizada para capacitar a los enfermeros en la práctica clínica y en futuras investigaciones.

## INTRODUCTION

Currently, the use of ultrasound (US) to measure bladder volume is a non-invasive, modern and fast resource in relation to the methods traditionally used^(1-4)^. In this regard, the use of US by a nurse at the bedside, point-of-care ultrasonography (POCUS), aims to increase the accuracy in clinical assessment performed by a nurse as well as greater safety in carrying out nursing interventions^(5)^. However, there are still few studies that address training nurses to use US as a foundation for professional practice^(6)^.

**Figure 1:**
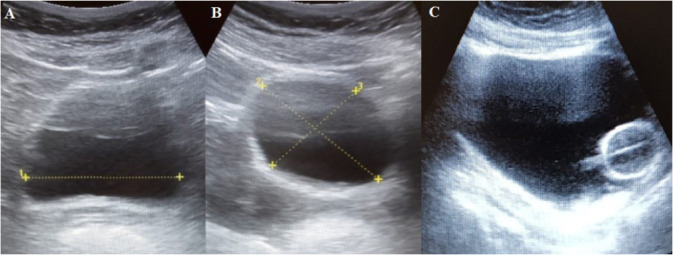
Analysis of the bladder transverse (A) and longitudinal (B) planes in the suprapubic region. Assessment of the correct catheter positioning inside the bladder by analyzing the catheter cuff (C).

Among the means for assessing bladder volume are anamnesis, physical examination, tomography and US^(7)^. The latter provides information regarding bladder interior assessment, where it is possible to assess the presence and quantity of fluids and, thus, calculate the intravesical volume through analysis of the transverse and longitudinal plane in the suprapubic region, as shown in Figure 1 ^(4)^. Given the ease in assessing bladder volume, the use of US has great applicability in a variety of patients, especially those who present risks of urinary retention^(1-7)^.

In addition to the ability to measure bladder volume, bladder US allows assessing the need for bladder catheterization (BC) or indwelling bladder catheterization (IBC), in order to avoid unnecessary procedures and, mainly, reduce the risk of urinary tract infections (UTI), in addition to the possibility of correctly assessing catheter positioning inside the bladder by analyzing the catheter cuff, as shown in Figure 1 ^(4,7)^. It is estimated that approximately 11% of patients using IBC develop UTI, and the length of time this catheter is in place is directly related to the increase in this incidence, in addition to generating an increase in hospital costs^(8,9)^. It has already been observed that, after the implementation of nurse training in POCUS for bladder volume control, there is a 20% reduction in UTI incidence associated with the bladder catheter^(10)^.

The use of POCUS by non-medical professionals has shown good results after a certain period of training^(11,12)^. In view of this, it is found that, after theoretical-practical training on POCUS, for the purpose of measuring bladder volume, a strong correlation is perceived between the volume found by a nurse in US and the volume of urine subsequently drained by bladder catheter^(2)^.

Despite the authorization for nurses to perform US in hospital and pre-hospital settings, as described in Resolution 679/2021^(5)^ of the Federal Nursing Council, this resolution does not list the minimum criteria for this training to be considered adequate^(5)^. Thus, it is necessary to delve deeper into this topic, since nursing has increasingly adopted technologies as a comprehensive part of advanced health practices, with the purpose of providing quality and safe care that directly impacts the length of hospital stay, outcome, patient satisfaction and costs related to care^(13)^.

Training in the use of US by nurses aims to develop competencies, which can be understood as an integration of knowledge, clinical judgment, skills, values and attitudes, indicating that the holistic competency view is widely accepted, since, in nursing practice, nurses must apply acquired knowledge, skills and innate individual characteristics to each situation and be able to adapt this knowledge and skills to different circumstances^(14)^.

Based on this competency framework, and with the development of new technologies in nursing clinical practice, especially the use of US, it is necessary to develop instruments that standardize the steps for carrying out the procedures, as well as the assessment of the knowledge and skills developed to carry out this intervention, and these instruments must be submitted to assessment by experts in the subject regarding the content so that all actions for carrying out the intervention are included.

Given the need to train nurses in the use of US and the scarcity of instruments that standardize the performance of these procedures and their consequent assessment of skills development, it is necessary to develop a checklist for this purpose. It is expected that, through the help of this checklist, it will be possible to carry out targeted and standardized training in an appropriate and safe manner, in order to obtain greater accuracy for training nurses to use this tool.

## OBJECTIVES

To develop and analyze evidence of content validity of a checklist for training nurses in measuring bladder volume through US.

## METHODS

### Ethical aspects

The study complied with Resolution 466/12 and Resolution 510/2016 of the Brazilian National Health Council, having been submitted to the institution’s Research Ethics Committee.

### Study design

This is a methodological study, referring to the construction and analysis of evidence of content validity of a checklist for training nurses in measuring bladder volume through US, carried out in three stages: literature review; instrument development; and analysis of evidence of content validity.

### Study stages

#### Narrative literature review

The first stage was to prepare an integrative literature review to find evidence to support the development of the proposed checklist items. Thus, according to criteria for developing a narrative literature review, the following guiding question was developed: what are the steps necessary to measure bladder volume using US?

Studies in Portuguese, English or Spanish, available in full, published between January 2010 and December 2020 (this time range is justified by the fact that the first studies on the subject were concentrated in this period), were included. The databases used were the US National Library of Medicine National Institutes of Health (PubMed), Latin American and Caribbean Literature in Health Sciences (LILACS), Scientific Electronic Library Online (SciELO) and Virtual Health Library (VHL). The choice of databases and virtual library is based on the number of indexed articles in health, databases that include primary studies as well as themes related to nursing.

The descriptors used for the search were selected according to the proposed theme, through the Health Sciences Descriptors (DECS) and the Medical Subject Heading (MESH). For the search strategy, the Boolean operator AND was used, and they were combined in different ways, with the aim of expanding the search for studies. The crosses performed with the descriptors were “*ultrassonografia*” AND “*bexiga vesical*”, “*ultrassonografia*” AND “*enfermagem*”, “*bexiga vesical*” AND “*enfermagem*”, “*ultrassonografia*” AND “*bexiga vesical*” AND “*enfermagem*”; “ultrassonography” AND “urinary bladder”, “ultrassonography” AND “nursing”, “urinary bladder” AND “nursing”.

The articles were selected by two researchers by reading titles and abstracts, and in case of doubts, one of the researchers with a PhD and experience in US assessed possible inconsistencies. Subsequently, the steps for assessing bladder volume described in articles were identified through full-text reading.

#### Checklist construction

After conducting the narrative literature review, the second stage consisted of developing items by identifying the fundamental steps for performing the technique of measuring bladder volume by US, focusing on nurses’ performance, based on evidence found in literature review. Hence, the items were developed considering factors intrinsic to patient safety, proper handling and maintenance of US equipment, and, finally, a step-by-step guide to measuring bladder volume. This classification within the checklist was performed based on the experience of two of the researchers in this study who have experience in US.

It is worth mentioning that the items are based on the criteria for structure, composition and naming of items from the Patient-Reported Outcomes Measurement Information System (PROMIS) Guideline^(15)^ on scientific standardization of instrument development and validity^(16)^. After constructing the checklist, each item was accompanied by a dichotomous response of the type “yes” and “no” regarding nurses’ performance of and item.

#### Analysis of evidence of content validity

Finally, the third stage consisted of assessing evidence of content validity of the items proposed by healthcare professionals (doctors and nurses) with experience in POCUS for assessing bladder volume with a minimum qualification of specialist in the area of activity (healthcare, teaching and/or research). Item assessment was carried out using the Delphi technique, which consists of a systematic method of judging information with the aim of obtaining consensus among experts on a given topic through validity articulated in phases or cycles^(16,17)^.

Initially, a group of experts was selected based on prior knowledge of the authors, following the inclusion criteria described above, and then an invitation to participate in the research was sent by email. Thus, upon acceptance, the Informed Consent Form (ICF) was applied digitally (through Google Forms^®^), and, subsequently, the checklist developed in the previous stage was sent.

In this way, experts assessed clarity (language used), relevance (association between the stage described in the checklist and existing theory) and dimensionality (whether each step contained in the checklist is related to its objective) of each proposed item, according to the definitions used^(15-17)^, using an agreement scale (-1 = do not agree with the maintenance of this item, 0 = partially agree with the maintenance of this item and +1 = agree with the maintenance of this item). For the items assessed as 0 or -1, suggestions for modification were requested, which were reformulated and submitted to a new round of assessment until consensus was reached.

#### Data analysis

The data were entered into the Research Electronic Data Capture (REDCap^®^) system^(18)^ by the researchers responsible for this study, in order to guarantee data security and anonymization. They were then imported into a Microsoft Excel^®^ 2007 spreadsheet. The analyses were performed using the Statistical Package for the Social Sciences (SPSS) version 22.0 and the R Development Core Team, both for Microsoft Windows^®^.

To analyze evidence of content validity, the Content Validity Index (CVI) was calculated using the formula: number of experts who scored +1, divided by the number of experts and multiplied by 100. For this index, values ≥0.80 were considered acceptable^(15,16)^.

The analysis of agreement among judges was performed using Gwet’s AC2 coefficient (second-order agreement coefficient), with a 95% Confidence Interval (95% CI) and a significance level of p≤0.050, with an agreement considered adequate when a coefficient greater than 0.80 was reached^(16)^. This test has been recommended as a way to reduce the limitations imposed by the Kappa agreement test^(17)^, characterized as a test that uses more than two judges with an ordered scale containing more than two categories^(17)^.

## RESULTS

**Figure 2:**
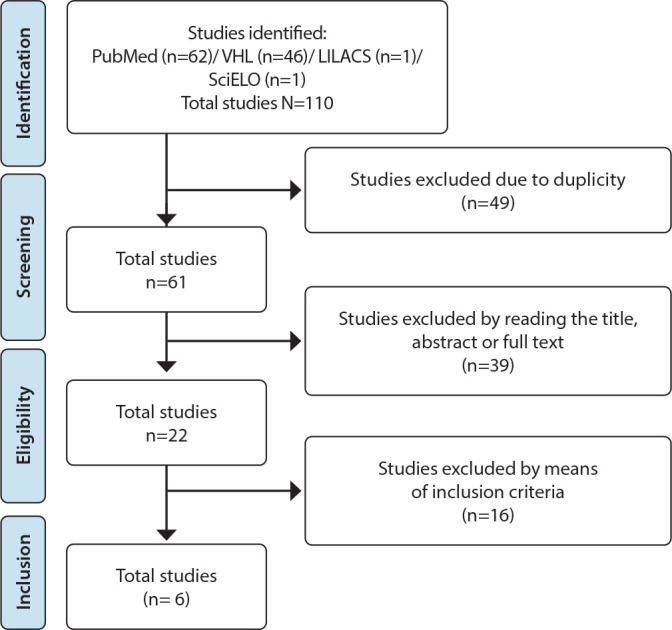
Process of inclusion of studies, São Paulo, São Paulo, Brazil, 2023

In the first stage of study, six articles were selected that contributed to developing the instrument. Figure 2 shows the study selection process.

*PubMed - US National Library of Medicine National Institutes of Health; VHL - Virtual Health Library; LILACS - Latin American and Caribbean Literature in Health Sciences; SciELO - Scientific Electronic Library Online virtual library.*

Six articles were identified, two of which were published in the United States, one in Italy, two in Brazil, and one in Japan. Regarding the year, one article was published in 2010, one in 2014, one in 2017, two in 2019, and one in 2020^(5,11,19-22)^. Of the articles included in the review, two developed technologies for training nurses^(9,21)^; an article developed a protocol for performing US to assess bladder volume^(22)^; an article developed a protocol for assessing urinary incontinence in patients after stroke using US as one of the assessment resources^(20)^; an article assessed students’ perception in urinary retention assessment using US and in performing bladder catheterization^(20)^; and an article assessed the scientific evidence for reducing urinary tract infection associated with bladder catheterization and reducing complications after training nurses in assessment using US^(11)^.

Thus, based on data identification in the literature, a checklist was developed for training and assessing nurses for measuring bladder volume through US, consisting of 23 items, which involved patient safety steps (identification and infection control measures), US device handling, transducer choice and configuration, location for image acquisition and the way of calculating residual bladder volume^(5,11,19-22)^.

After developing the checklist, it was sent to ten judges, consisting of nine nurses with more than five years of training, with experience in using US to measure bladder residue volume and experience with methodological studies, and an intensive care physician with experience in US.

Table 1 shows the values achieved in relation to the CVI for each indicator assessed. In the clarity criterion, 21 maximum scores were obtained (100.0%). In the relevance criterion, 22 agreement values were observed at 100.0%. In the dimensionality criterion, 20 maximum scores were obtained (100.0%). No item received a score lower than 80.0% and, therefore, all were maintained at this stage.

**Table 1:** Content Validity Index regarding item adequacy in relation to the criteria of clarity, relevance and dimensionality, São Paulo, São Paulo, Brazil, 2023

Checklist items	Clarity	Relevance	Dimensionality
Performs hand hygiene.	100.0	100.0	100.0
Identifies patient.	100.0	100.0	100.0
Explains the procedure to patient.	100.0	100.0	100.0
Places patient in a horizontal dorsal decubitus position.	100.0	100.0	100.0
Turns on the device and inserts patient’s identification.	100.0	100.0	100.0
Chooses the appropriate transducer (convex) to view the bladder.	100.0	100.0	100.0
Applies the gel to the transducer reader.	100.0	100.0	100.0
Holds the transducer properly (holds the transducer with the index finger and thumb, and rests it on the middle finger).	100.0	100.0	90.0
Positions the transducer properly in the suprapubic region in the transverse plane.	100.0	100.0	100.0
Scans (locates) the bladder properly in the transverse plane.	100.0	100.0	100.0
Measures the bladder in the transverse plane (side-to-side).	100.0	100.0	100.0
Saves the value found.	100.0	100.0	100.0
Properly positions the transducer in the suprapubic region in the sagittal plane.	100.0	100.0	100.0
Properly scans (locates) the bladder in the sagittal plane.	100.0	100.0	100.0
Measures the bladder in the sagittal plane (in the superior-inferior angle and anteroposterior angle).	100.0	100.0	100.0
Saves the values found.	100.0	100.0	100.0
Identifies the bladder volume.	90.0	100.0	90.0
Correlates ultrasound findings (bladder residue and indwelling bladder catheterization balloon) with patient’s clinical condition.	90.0	80.0	80.0
Clean the gel from the suprapubic region with paper or a towel.	100.0	100.0	100.0
Place patient in a comfortable position.	100.0	100.0	100.0
Disinfect ultrasound device and transducer with disinfectant/degermer.	100.0	100.0	100.0
Perform hand hygiene.	100.0	100.0	100.0
Make a note of the procedure performed.	100.0	100.0	100.0
Total (%)	99.1	99.1	98.3

When assessing agreement among judges using Gwet’s AC2 coefficient, excellent agreement was identified among evaluators in all indicators assessed, as shown in Table 2.

**Table 2:** Inter-judge agreement coefficients, São Paulo, São Paulo, Brazil, 2023

Aspect	Coefficient (95% CI)^*^	*p* value†
Clarity	0.89 (0.84; 0.95)	<0.001
Relevance	0.97 (0.94; 0.99)	<0.001
Dimensionality	0.95 (0.91; 0.99)	<0.001

* *Confidence Interval; †Gwet’s AC2 test.*

Even though the CVI value was higher than the critical value considered adequate, some suggestions from reviewers were accepted, such as including guidance for the companion, replacing the word “place” with “position”, including the word “register” in the action of saving the data and including the word “calculate” in the action of identifying the residual bladder volume. Chart 1 displays the final version of the checklist for training nurses to measure bladder residue by US.

**Chart 1:** Final version of a training checklist for nurses to measure bladder volume by ultrasound, São Paulo, São Paulo, Brazil, 2023

Steps		Action taken
1.	Performs hand hygiene.	( ) Yes ( ) No
2.	Identifies patient.	( ) Yes ( ) No
3.	Explains the procedure to patient and/or companion.	( ) Yes ( ) No
4.	Properly positions patient in horizontal dorsal decubitus.	( ) Yes ( ) No
5.	Turns on the device and inserts patient’s identification.	( ) Yes ( ) No
6.	Selects the appropriate transducer for viewing the bladder (convex transducer).	( ) Yes ( ) No
7.	Applies the gel to the transducer reader.	( ) Yes ( ) No
8.	Properly holds the transducer (holds the transducer with the index finger and thumb, and rests it on the middle finger).	( ) Yes ( ) No
9.	Properly positions the transducer in the suprapubic region in the transverse plane.	( ) Yes ( ) No
10.	Scans and locates the bladder appropriately in the transverse plane.	( ) Yes ( ) No
11.	Measures the bladder in the transverse plane (lateral-lateral).	( ) Yes ( ) No
12.	Save/records the value found.	( ) Yes ( ) No
13.	Properly positions the transducer in the suprapubic region in the sagittal plane.	( ) Yes ( ) No
14.	Scans and locates the bladder appropriately in the sagittal plane.	( ) Yes ( ) No
15.	Measures the bladder in the sagittal plane (in the superior-inferior axis and anteroposterior axis).	( ) Yes ( ) No
16.	Save/records the values found.	( ) Yes ( ) No
17.	Identifies/calculates bladder volume.	( ) Yes ( ) No
18.	Correlates ultrasound findings (bladder volume and indwelling bladder catheterization balloon) with patient’s clinical condition.	( ) Yes ( ) No
19.	Cleans the suprapubic region appropriately.	( ) Yes ( ) No
20.	Positions patient appropriately.	( ) Yes ( ) No
21.	Disinfects the ultrasound device and the transducer used.	( ) Yes ( ) No
22.	Performs hand hygiene.	( ) Yes ( ) No
23.	Record and write down the procedure performed.	( ) Yes ( ) No

## DISCUSSION

There is a growing interest in adopting practices capable of innovating and reforming health systems to respond to problems arising from populations’ health needs, especially due to the increase in chronic conditions, with the incorporation of technologies being one of these advances, especially those that can increase safety in nursing care, bringing greater accuracy in nurses’ clinical assessment and greater assertiveness in relation to nursing procedures^(23,24)^. It was within this premise that this study developed and achieved adequate evidence of content validity of a checklist that aimed to standardize the steps for assessing bladder volume by US as well as assessing professionals’ adherence to the technique and consequent technique assessment.

In order to perform bladder US, it is necessary to implement educational programs aimed at training nurses for this practice. In a study conducted with 38 nurses for training in performing POCUS to assess bladder volume, it was found that 93% of nurses after the educational program responded correctly about the basic knowledge of US and demonstrated high agreement in residual bladder volume assessment^(6)^.

Training programs initially require constructing instruments that can guide educational training towards the development of expected competencies involving the acquisition of knowledge and development of skills^(14)^. These instruments, which aim to standardize procedures and assess the development of competencies, have as their main objective to guide the implementation of the technique so that it can be carried out without harm^(12)^.

It is worth noting that evidence on checklist validity for training nurses to use POCUS is scarce. Among the studies that demonstrate nurse training in POCUS, in general, there is information on the training content, such as theoretical classes on the basic components of US, the type of validity with US and the results found after training^(4,6,12,25)^. However, there is no instrument that guides the steps in the bedside verification process using US in order to find the necessary structures to compose nurses’ reasoning regarding venipuncture, bladder volume, gastric content and volume, and vascular and pulmonary assessment^(4,6,12,25)^.

The use of procedural checklists can reduce professionals’ dependence on memory, reduce errors, and assist in skills development assessment, based on a low-cost resource for health services^(27)^, and can be developed based on the necessary professional skills goals. Instruments based on skills development and assessment have been recommended, since they take into account several aspects of professional performance, ranging from professionals’ behavior to the implementation of evidence-based practices, concern for patient safety aspects, use of information systems, and incorporation of technologies in clinical care practice^(27)^.

The construction of this checklist was divided into three main domains, which are steps involving patient safety (patient identification, communication with patient and family and recording of identified data), general handling of US equipment (correct choice of transducer and its configuration, application of conductive gel, way of handling the transducer) and correct image acquisition for measuring bladder volume^(4,11,19-22)^.

After the checklist was constructed, it was assessed by a group of ten experts in US in relation to its content. This form of analysis of validity evidence is essential to provide data on the degree to which the elements of an assessment instrument/checklist are relevant and representative of the main construct and are clear as to their action. The representativeness of the group of experts in terms of number and professional training is another important factor in content validity studies, and the group of experts in this study had extensive experience in US^(28)^.

For analysis of evidence of content validity, CVI calculation was chosen, as it is the most commonly used statistical calculation in this type of assessment, and the CVI values achieved in this study exceeded the cut-off point established in the literature of 80%^(16)^. Another statistical calculation adopted in this study was the analysis of agreement among experts, using Gwet’s second-order coefficient of agreement among judges (Gwet’s AC2), which can be used when there are two or more judges with a classification scale with two or more categories, and the closer to 1, the lower the probability of agreement among judges occurring due to chance. In the present study, the coefficient was higher than the established cut-off point (0.80)^(17)^. The null hypothesis for Gwet’s AC2, as for other measures of agreement, is that there is no agreement beyond what would be expected by chance. A high AC2 value indicates significant agreement among judges, suggesting that the null hypothesis can be rejected^(29)^.

Therefore, the creation of a checklist for the current study becomes essential to guide the process of training nurses at the bedside with regard to identifying the main fundamental steps for verifying bladder volume through US. The need for new studies that address the application of this checklist for training nurses and its implications for these professionals’ accuracy in using the POCUS tool to verify bladder volume stands out.

### Study limitations

This study developed a checklist for assessing bladder volume by US, but did not perform a pre-test for its application nor its usefulness in skills development, with this gap being identified as a limitation of this study.

### Contributions to nursing

The checklist developed to assess skills for performing US to measure bladder volume can be applied in educational processes and nurse training, as it provides standardization in the assessment of the skills developed.

## CONCLUSIONS

The checklist developed through an integrative literature review to assess skills development for measuring bladder volume by US was composed of 23 items and divided into three domains: steps involving patient safety; general handling of US equipment; and correct image acquisition for measuring bladder volume. The checklist achieved adequate evidence of content validity, with CVI greater than 0.80 in all items, and a high level of agreement among evaluators, and can be used in educational practice at different levels of nursing training.
