# Supplementary material for: Bladder ultrasound: evidence of content validity of a checklist for training nurses
Source: Rev Bras Enferm. 2024 Dec 16;77(6):e20230183. doi: 10.1590/0034-7167-2023-0183 (PMC11654563; doi:10.1590/0034-7167-2023-0183)
Supplement: Translation

# Translation: Ultrassonografia vesical: evidências de validade de conteúdo de um *checklist* para capacitação de enfermeiros

**DOI:** 10.1590/0034-7167-2023-0183pt

**Authors:** Filipe Utuari de Andrade Coelho, Sabrina Martins Reigota, Flávia Manfredi Cavalcanti, Dejanira Aparecida Regagnin, Beatriz Murata Murakami, Vinícius Batista Santos

**Affiliations:** I Universidade de São Paulo. São Paulo, São Paulo, Brasil; II Hospital Israelita Albert Einstein. São Paulo, São Paulo, Brasil; III Hospital DF Star. Brasília, Distrito Federal, Brasil; IV Universidade Federal de São Paulo. São Paulo, São Paulo, Brasil

**Keywords:** Ultrassom, Estudos de Validação, Bexiga Urinária, Enfermagem, Prática Avançada de Enfermagem.

## Abstract

**Objetivos:**

desenvolver e analisar as evidências de validade de conteúdo de um *checklist* para capacitação de enfermeiros na mensuração do volume vesical através da ultrassonografia.

**Métodos:**

estudo metodológico, constituído por três etapas: revisão da literatura; elaboração dos itens do instrumento; e análise das evidências de validade de conteúdo. O Índice de Validade de Conteúdo (IVC) e o AC2 de Gwet foram utilizados para as análises da validade de conteúdo.

**Resultados:**

o *checklist* foi composto por 23 itens. Os IVCs para clareza, relevância e dimensionalidade foram 0,99, 0,99 e 0,98 respectivamente, e os IVCs para AC2 de Gwet com coeficientes para clareza, relevância e dimensionalidade foram 0,89, 0,97 e 0,95, respectivamente, com *p*<0,001.

**Conclusões:**

o *checklist* desenvolvido para capacitação de enfermeiros na mensuração do volume vesical através da ultrassonografia alcançou adequadas evidências de validade de conteúdo, e pode ser utilizado para capacitação dos enfermeiros na prática clínica e futuras pesquisas.

## INTRODUÇÃO

Atualmente, a utilização da ultrassonografia (USG) para mensurar o volume vesical é um recurso não invasivo, moderno e rápido em relação aos métodos tradicionalmente empregados^(1-4)^. Nesse sentido, a utilização da USG pelo enfermeiro a beira do leito, *point-of-care ultrasonography* (POCUS), visa aumentar a acurácia na avaliação clínica realizada pelo enfermeiro, bem como maior segurança na realização de intervenções de enfermagem^(5)^. Contudo, ainda são escassos estudos que abordem a capacitação dos enfermeiros para a utilização da USG como alicerce para a prática profissional^(6)^.

Entre os meios para avaliação do volume vesical, estão a anamnese, o exame físico, a tomografia e a USG^(7)^. Este último fornece informações a respeito da avaliação do interior vesical, onde é possível avaliar a presença e a quantidade de líquidos e, assim, realizar o cálculo do volume intravesical por meio da análise do plano transversal e longitudinal na região suprapúbica, conforme Figura 1 ^(4)^. Diante da facilidade na avaliação do volume vesical, a utilização da USG tem grande aplicabilidade em uma variedade de pacientes, especialmente aqueles que apresentam riscos de retenção urinária^(1-7)^.

**Figura 1:** Análise do plano transversal (A) e longitudinal (B) da bexiga na região suprapúbica. Avaliação do posicionamento correto do cateter no interior da bexiga por meio da análise do balonete do cateter (C)

Além da capacidade da mensuração do volume vesical, a USG da bexiga permite avaliar a necessidade de cateterismo vesical de alívio (CVA) ou cateterismo vesical de demora (CVD), de modo a evitar procedimentos desnecessários e, principalmente, reduzir o risco de infeções do trato urinário (ITU), além da possibilidade da avaliação correta do posicionamento do cateter no interior da bexiga por meio da análise do balonete do cateter, conforme a Figura 1 ^(4,7)^. Estima-se que cerca de 11% dos pacientes em uso de CVD desenvolvam ITU, e o tempo de permanência deste cateter está diretamente relacionado ao aumento desta incidência, além de gerar aumento nos custos hospitalares^(8,9)^. Já é observado que, após a implementação do treinamento de enfermeiros em POCUS para controle de volume vesical, ocorre uma redução de 20% na incidência de ITU associada ao cateter vesical^(10)^.

A utilização do POCUS por profissionais não médicos tem apresentado bons resultados após determinado período de treinamento^(11,12)^. Diante disso, verifica-se que, posteriormente ao treinamento teórico-prático sobre POCUS, para fins de mensuração do volume vesical, é percebida uma forte correlação entre volume encontrado pelo enfermeiro na USG e o volume de urina drenado subsequente por cateter vesical^(2)^.

Apesar da autorização para a realização da USG por enfermeiros no âmbito hospitalar e pré-hospitalar, conforme descrito na Resolução n^o^ 679/2021^(5)^ do Conselho Federal de Enfermagem, não são elencados nesta resolução os critérios mínimos para que essa capacitação seja considerada adequada^(5)^. Assim, faz-se necessário um aprofundamento nesta temática, uma vez que a enfermagem tem se apropriado cada vez mais das tecnologias como parte integrante das práticas avançadas em saúde, com o propósito de empregar uma assistência de qualidade e segura que impacte diretamente o tempo de internação hospitalar, desfecho, satisfação do paciente e custos relacionados à assistência^(13)^.

O treinamento do uso da USG por enfermeiros visa ao desenvolvimento de competências, que pode ser entendida como uma integração de conhecimento, julgamento clínico, habilidades, valores e atitudes, indicando que a visão de competência holística é amplamente aceita, pois, na prática de enfermagem, os enfermeiros devem aplicar os conhecimentos adquiridos, as habilidades e as características individuais inatas a cada situação e ser capaz de adaptar esse conhecimento e essas habilidades para circunstâncias diferentes^(14)^.

Com base neste referencial de competências, e com o desenvolvimento de novas tecnologias na prática clínica do enfermeiro, em especial o uso da USG, faz-se necessário o desenvolvimento de instrumentos que padronizem os passos para a realização dos procedimentos, bem como a avaliação dos conhecimentos e habilidades desenvolvidas para a realização dessa intervenção, e esses instrumentos devem ser submetidos à avaliação de especialistas na temática quanto ao conteúdo, para que todas as ações para a realização da intervenção estejam incluídas.

Diante da necessidade de treinamento dos enfermeiros para o uso da USG e da escassez de instrumentos que padronizem a realização desses procedimentos e sua consequente avaliação de desenvolvimento de competências, faz-se necessário o desenvolvimento de um *checklist* para este fim. Espera-se que, através do auxílio desse *checklist*, seja possível realizar um treinamento direcionado e padronizado de forma adequada e segura, a fim de se obter uma melhor acurácia no treinamento dos enfermeiros no manuseio dessa ferramenta.

## OBJETIVOS

Desenvolver e analisar as evidências de validade de conteúdo de um *checklist* para capacitação de enfermeiros na mensuração do volume vesical através da USG.

## MÉTODOS

### Aspectos éticos

O estudo atendeu à Resolução n^o^ 466/12 e à Resolução n^o^ 510/2016 do Conselho Nacional de Saúde, tendo sido submetido ao Comitê de Ética em Pesquisa da instituição.

### Tipo de estudo

Trata-se de estudo do tipo metodológico, referente à construção e análise das evidências de validade de conteúdo de um *checklist* para capacitação de enfermeiros na mensuração de volume vesical através da USG, realizado em três etapas: revisão da literatura; desenvolvimento do instrumento; e análise das evidências de validade de conteúdo.

### Etapas do estudo

#### Revisão narrativa de literatura

A primeira etapa foi a elaboração de revisão integrativa de literatura, a fim de encontrar evidências que embasassem a elaboração dos itens do *checklist* proposto. Dessa maneira, de acordo com os critérios para elaboração de revisão narrativa de literatura, foi elaborada a seguinte questão norteadora: quais as etapas necessárias para realização da mensuração do volume vesical utilizando a USG?

Publicações em português, inglês ou espanhol, disponíveis na íntegra, publicadas entre janeiro de 2010 e dezembro de 2020 (esta faixa temporal se justifica devido ao surgimento das primeiras publicações a respeito do tema estar concentrado neste período), foram incluídas. As bases de dados utilizadas foram *US National Library of Medicine National Institutes of Health* (PubMed), Literatura Latino-Americana e do Caribe em Ciências da Saúde (LILACS), biblioteca virtual *Scientific Eletronic Library Online* (SciELO) e Biblioteca Virtual em Saúde (BVS). A escolha das bases de dados e da biblioteca virtual baseia-se no quantitativo de indexação de artigos da área da saúde, bases que contemplam estudos primários, bem como temáticas relacionadas à enfermagem.

Os descritores utilizados para busca foram selecionados de acordo com o tema proposto, por meio dos Descritores em Ciências da Saúde (DECS) e do *Medical Subject Heading* (MESH). Para a estratégia de busca, foi utilizado o operador booleano *AND,* e foram combinados de diferentes maneiras, com o objetivo de ampliar a busca pelos estudos. Os cruzamentos realizados com os descritores foram “ultrassonografia” *AND* “bexiga vesical”, “ultrassonografia” *AND* “enfermagem”, “bexiga vesical” *AND* “enfermagem”, “ultrassonografia” *AND* “bexiga vesical” *AND* “enfermagem”; “*ultrassonography” AND* “*urinary bladder”,* “*ultrassonography” AND* “*nursing*”, “*urinary bladder” AND* “*nursing*”.

Os artigos foram selecionados por dois pesquisadores por meio da leitura do título e resumo, e, em caso de dúvidas, um dos pesquisadores com titulação de doutor em ciências e experiência em USG avaliou as possíveis inconsistências. Posteriormente, foram identificados, por meio da leitura completa, os passos para a realização da avaliação do volume vesical descrito nos artigos.

#### Construção do checklist

Após a realização da revisão narrativa de literatura, a segunda etapa consistiu em desenvolver os itens através da identificação dos passos fundamentais para realização da técnica de mensuração do volume vesical pela USG, com foco na atuação do enfermeiro, baseados pelas evidências encontradas na revisão de literatura. Nesse sentido, os itens foram desenvolvidos considerando fatores intrínsecos à segurança do paciente, ao manuseio e à manutenção adequada do equipamento de ultrassom, e, por fim, sobre o passo a passo de como mensurar o volume vesical. Esta classificação dentro do *checklist* foi realizada com base na experiência de dois dos pesquisadores deste estudo que possuem experiência na área de USG.

Vale ressaltar que os itens estão fundamentados pelos critérios de estrutura, composição e nomeação de itens do *Guideline Patient-Reported Outcomes Measurement Information System* (PROMIS)^(15)^ sobre padronização científica do desenvolvimento e validação de instrumentos^(16)^. Após a construção do *checklist*, cada item foi acompanhado de resposta dicotômica do tipo “sim” e “não” referente à realização do item pelo enfermeiro.

#### Análise das evidências de validade de conteúdo

Por fim, a terceira etapa consistiu na avaliação das evidências de validade de conteúdo dos itens propostos por profissionais da área da saúde (médico e enfermeiro) com experiência na área de POCUS para avaliação do volume vesical com titulação mínima de especialista na área de atuação (assistência, ensino e/ou pesquisa). A avaliação dos itens se deu por meio da técnica Delphi, a qual consiste em método sistematizado de julgamento de informações com o intuito de se obter consensos de especialistas sobre determinado tema por meio de validações articuladas em fases ou ciclos^(16,17)^.

Inicialmente, foi selecionado um grupo de especialistas por conhecimento prévio dos autores seguindo os critérios de inclusão descritos acima, e a seguir foi enviado um convite de participação na pesquisa por *e-mail*. Assim, após o aceite, o Termo de Consentimento Livre e Esclarecido (TCLE) foi aplicado por meio digital (através da plataforma *Google Forms^®^*), e, posteriormente, foi enviado o *checklist* desenvolvido na etapa anterior.

Dessa maneira, os especialistas avaliaram a clareza (linguagem utilizada), a relevância (associação entre o passo descrito no *checklist* e a teoria existente) e a dimensionalidade (se cada passo contido no *checklist* está relacionado ao objetivo do mesmo) de cada item proposto, conforme definições utilizadas^(15-17)^, utilizando uma escala de concordância (-1= não concordo com a manutenção do item, 0= concordo parcialmente com a manutenção do item e +1= concordo com a manutenção do item). Para os itens avaliados como 0 ou -1, foram solicitadas sugestões de modificação, sendo reformulados e submetidos à nova rodada de avaliação até a obtenção do consenso.

#### Análise dos dados

Os dados foram inseridos no sistema *Research Electronic Data Capture* (REDCap^®^)^(18)^ pelos pesquisadores responsáveis deste estudo, com o intuito de garantir a segurança e a anonimização dos dados. Posteriormente, foram importados para a planilha eletrônica do programa *Microsoft Excel* ^®^ 2007. As análises foram realizadas com o auxílio do programa *software Statistical Package for the Social Sciences* (SPSS), versão 22.0, e do programa R *Development Core Team*, ambos para o *Microsoft Windows* ^®^.

Para análise das evidências de validade de conteúdo, foi calculado o Índice de Validade de Conteúdo (IVC) pela fórmula: número de especialistas que pontuaram o escore +1, dividido pelo número de especialistas e multiplicado por 100. Para este índice, foram considerados aceitáveis valores ≥0,80^(15,16)^.

A análise de concordância entre os juízes foi realizada pelo coeficiente AC2 de Gwet (*second-order agreement coefficient*), com Intervalo de Confiança de 95% (IC 95%) e nível de significância de *p*≤0,050, sendo considerada uma concordância adequada quando atingido um coeficiente superior a 0,80^(16)^. Este teste tem sido recomendado como uma forma de reduzir as limitações impostas pelo teste de concordância de Kappa^(17)^, caracterizando-se como um teste que utiliza mais de dois juízes com uma escala ordenada contendo mais de duas categorias^(17)^.

## RESULTADOS

Na primeira etapa do estudo, selecionaram-se seis artigos que contribuíram para elaboração do instrumento. O processo de seleção dos estudos está na Figura 2.

**Figura 2:** Processo de inclusão dos estudos, São Paulo, São Paulo, Brasil, 2023

*PubMed - US National Library of Medicine National Institutes of Health; BVS - Biblioteca Virtual em Saúde; LILACS - Literatura Latino-Americana e do Caribe em Ciências da Saúde; SciELO - biblioteca virtual Scientific Eletronic Library Online.*

Foram identificados seis artigos, sendo que dois foram realizados nos Estados Unidos, um, na Itália, dois, no Brasil, e um, no Japão. Em relação ao ano, um artigo foi publicado em 2010, um, em 2014, um, em 2017, dois, em 2019, e um, em 2020^(5,11,19-22)^. Dos artigos incluídos na revisão, dois desenvolveram tecnologias para treinamento dos enfermeiros^(9,21)^; um artigo desenvolveu um protocolo para a realização da USG para avaliação do volume vesical^(22)^; um artigo desenvolveu um protocolo para avaliação de incontinência urinária em pacientes após acidente vascular encefálico (AVE) utilizando a USG como um dos recursos de avaliação^(20)^; um artigo avaliou a percepção dos estudantes na avaliação da retenção urinária utilizando a USG e na realização da cateterização vesical^(20)^; e um artigo avaliou as evidências científicas para redução da infecção urinária associada ao cateterismo vesical e à redução de complicações após o treinamento dos enfermeiros na avaliação por meio da USG^(11)^.

Assim, a partir da identificação dos dados na literatura, foi desenvolvido o *checklist* para capacitação e avaliação dos enfermeiros na mensuração de volume vesical através da USG composto por 23 itens, que envolveram etapas da segurança do paciente (identificação e medidas de controle de infecção), manipulação do aparelho de USG, escolha e configuração do transdutor, local para aquisição da imagem e a forma de realização do cálculo do volume residual vesical^(5,11,19-22)^.

Após o desenvolvimento do *checklist*, o mesmo foi enviado para dez juízes, composto por nove enfermeiros com tempo de formação superior a cinco anos, com experiência no uso da USG para mensuração do volume de resíduo vesical e experiência com estudos metodológicos e um médico intensivista com experiência em USG.

A Tabela 1 demonstra os valores alcançados em relação ao IVC para cada indicador avaliado. No critério de clareza, foram obtidos 21 escores máximos (100,0%). Já no critério de relevância, foram observados 22 valores de concordância em 100,0%. No critério de dimensionalidade, foram obtidos 20 escores máximos (100,0%). Nenhum item recebeu escore menor que 80,0% e, portanto, todos foram mantidos nesta etapa.

**Tabela 1:** Índice de Validade de Conteúdo quanto à adequação dos itens em relação aos critérios de clareza, relevância e dimensionalidade, São Paulo, São Paulo, Brasil, 2023

Itens do *checklist*	Clareza	Relevância	Dimensionalidade
Realiza a higiene das mãos.	100,0	100,0	100,0
Identifica o paciente.	100,0	100,0	100,0
Explica o procedimento para o paciente.	100,0	100,0	100,0
Coloca o paciente em decúbito dorsal horizontal.	100,0	100,0	100,0
Liga o aparelho e insere a identificação do paciente.	100,0	100,0	100,0
Escolhe o transdutor adequado (convexo) para visualizar a bexiga.	100,0	100,0	100,0
Aplica o gel no leitor do transdutor.	100,0	100,0	100,0
Segura adequadamente o transdutor (segura o transdutor com o dedo indicador e o polegar, e o apoia sobre o dedo médio).	100,0	100,0	90,0
Posiciona adequadamente o transdutor na região suprapúbica no plano transversal.	100,0	100,0	100,0
Realiza a varredura (localiza) da bexiga adequadamente no plano transversal.	100,0	100,0	100,0
Realiza a medição da bexiga no plano transversal (latero-lateral).	100,0	100,0	100,0
Salva o valor encontrado.	100,0	100,0	100,0
Posiciona adequadamente o transdutor na região suprapúbica no plano sagital.	100,0	100,0	100,0
Realiza a varredura (localiza) da bexiga adequadamente no plano sagital.	100,0	100,0	100,0
Realiza a medição da bexiga no plano sagital (em ângulo superior-inferior e ângulo ântero-posterior).	100,0	100,0	100,0
Salva os valores encontrados.	100,0	100,0	100,0
Identifica o volume vesical.	90,0	100,0	90,0
Correlaciona o achado da ultrassonografia (resíduo vesical e balonete do cateterismo vesical de demora) com a clínica do paciente.	90,0	80,0	80,0
Realiza a limpeza do gel da região suprapúbica com papel ou toalha.	100,0	100,0	100,0
Coloca o paciente em posição confortável.	100,0	100,0	100,0
Realiza a desinfecção do aparelho de ultrassonografia e do transdutor com desinfectante/degermante.	100,0	100,0	100,0
Realiza higiene das mãos.	100,0	100,0	100,0
Realiza a anotação do procedimento realizado.	100,0	100,0	100,0
Total (%)	99,1	99,1	98,3

Na avaliação da concordância entre os juízes por meio do coeficiente AC2 de Gwet, foi identificada uma concordância excelente entre os avaliadores em todos os indicadores avaliados, conforme Tabela 2.

**Tabela 2:** Coeficientes de concordância entre os juízes, São Paulo, São Paulo, Brasil, 2023

Aspecto	Coeficiente (IC 95%)^*^	Valor de *p*†
Clareza	0,89 (0,84; 0,95)	<0,001
Relevância	0,97 (0,94; 0,99)	<0,001
Dimensionalidade	0,95 (0,91; 0,99)	<0,001

* *Intervalo de Confiança; † teste AC2 de Gwet.*

Mesmo tendo sido obtido um valor de IVC superior ao valor crítico considerado adequado, foram acatadas algumas sugestões dos revisores, como incluir a orientação ao acompanhante, substituição da palavra “coloca” por “posiciona”, incluir a palavra “registrar” na ação de salvar os dados e incluir a palavra “calcula” na ação de identificar o volume residual vesical. A versão final do *checklist* para capacitação dos enfermeiros para mensuração do resíduo vesical pela USG está disposto no Quadro 1.

**Quadro 1:** Versão final do *checklist* de capacitação dos enfermeiros para mensuração do volume vesical pela ultrassonografia, São Paulo, São Paulo, Brasil, 2023

Passos		Ação realizada
1.	Realiza higiene das mãos.	( ) Sim ( ) Não
2.	Identifica o paciente.	( ) Sim ( ) Não
3.	Explica o procedimento ao paciente e/ou acompanhante.	( ) Sim ( ) Não
4.	Posiciona adequadamente o paciente em decúbito dorsal horizontal.	( ) Sim ( ) Não
5.	Liga o aparelho e insere a identificação do paciente.	( ) Sim ( ) Não
6.	Seleciona o transdutor adequado para visualização da bexiga (transdutor convexo).	( ) Sim ( ) Não
7.	Aplica o gel no leitor do transdutor.	( ) Sim ( ) Não
8.	Segura adequadamente o transdutor (segura o transdutor com o dedo indicador e o polegar, e o apoia sobre o dedo médio).	( ) Sim ( ) Não
9.	Posiciona adequadamente o transdutor na região suprapúbica no plano transversal.	( ) Sim ( ) Não
10.	Realiza a varredura e localiza a bexiga adequadamente no plano transversal.	( ) Sim ( ) Não
11.	Realiza a medição da bexiga no plano transversal (latero-lateral).	( ) Sim ( ) Não
12.	Salva/registra o valor encontrado.	( ) Sim ( ) Não
13.	Posiciona adequadamente o transdutor na região suprapúbica no plano sagital.	( ) Sim ( ) Não
14.	Realiza a varredura e localiza a bexiga adequadamente no plano sagital.	( ) Sim ( ) Não
15.	Realiza a medição da bexiga no plano sagital (em eixo superior-inferior e eixo ântero-posterior).	( ) Sim ( ) Não
16.	Salva/registra os valores encontrados.	( ) Sim ( ) Não
17.	Identifica/calcula o volume vesical.	( ) Sim ( ) Não
18.	Correlaciona o achado da ultrassonografia (volume vesical e balonete do cateterismo vesical de demora) com a clínica do paciente.	( ) Sim ( ) Não
19.	Realiza a limpeza da região suprapúbica adequadamente.	( ) Sim ( ) Não
20.	Posiciona o paciente adequadamente.	( ) Sim ( ) Não
21.	Realiza a desinfecção do aparelho de ultrassonografia e do transdutor utilizado.	( ) Sim ( ) Não
22.	Realiza higiene das mãos.	( ) Sim ( ) Não
23.	Registra e anota o procedimento realizado.	( ) Sim ( ) Não

## DISCUSSÃO

Observa-se crescente interesse em adotar práticas capazes de inovar e reformar sistemas de saúde para responder aos problemas decorrentes das necessidades de saúde das populações, especialmente em função do aumento de condições crônicas, sendo a incorporação de tecnologias um desses avanços, em especial aquelas que possam aumentar a segurança nos cuidados de enfermagem, trazendo maior acurácia na avaliação clínica do enfermeiro e maior assertividade em relação aos procedimentos de enfermagem^(23,24)^. Foi dentro dessa premissa que este estudo desenvolveu e alcançou adequadas evidências de validade de conteúdo de um *checklist* que teve como objetivo padronizar os passos para a realização da avaliação do volume vesical pela USG, bem como avaliar a aderência do profissional à técnica e consequente avaliação da técnica.

Para a realização da USG vesical, é necessário a implementação de programas educacionais visando à capacitação dos enfermeiros para esta prática. Em estudo realizado com 38 enfermeiros para capacitação na realização do POCUS para avaliação do volume vesical, identificou-se que 93% dos enfermeiros após o programa educacional responderam corretamente sobre o conhecimento básico da USG e demonstraram elevada concordância na avaliação do volume residual vesical^(6)^.

Os programas de treinamento necessitam, inicialmente, da construção de instrumentos que possam guiar a formação educacional para o desenvolvimento das competências esperadas que envolvem a aquisição de conhecimento e desenvolvimento de habilidades^(14)^. Esses instrumentos que visam padronizar os procedimentos e avaliar o desenvolvimento de competências têm como objetivo principal nortear a implementação da técnica para que possa ser realizada livre de danos^(12)^.

Vale ressaltar que evidências sobre a validação de *checklist* para capacitação de enfermeiros para utilização do POCUS são escassas. Entre os estudos que evidenciam capacitação de enfermeiros em POCUS, de forma geral, há informações sobre o conteúdo do treinamento, como aulas teóricas sobre os componentes básicos de USG, o tipo de validação com o USG e os resultados encontrados após a capacitação^(4,6,12,25)^. Contudo, não há instrumento que guie as etapas no processo de verificação à beira leito no uso da USG a fim de se encontrar as estruturas necessárias para compor o raciocínio do enfermeiro no que tange à punção venosa, ao volume vesical, ao conteúdo e volume gástrico e à avaliação vascular e pulmonar^(4,6,12,25)^.

O uso de *checklists* de procedimentos pode reduzir a dependência da memória do profissional, reduzir erros, além de auxiliar na avaliação do desenvolvimento das competências, baseado em um recurso de baixo custo aos serviços de saúde^(26)^, e podem ser elaborados baseados nas metas de competências profissionais necessárias. Os instrumentos baseados em desenvolvimento e avaliação de competência têm sido recomendados, uma vez que levam em consideração diversos aspectos da atuação profissional, que vão desde o comportamento do profissional até a implementação das práticas baseadas em evidências, preocupação com os aspectos da segurança do paciente, utilização dos sistemas de informação e incorporação de tecnologias na prática clínica assistencial^(27)^.

A construção deste *checklist* foi dividido em três domínios principais, sendo eles passos envolvendo a segurança do paciente (identificação do paciente, comunicação com o paciente e família e registro dos dados identificados), manuseio geral dos aparelhos de USG (escolha correta do transdutor e de sua configuração, aplicação do gel condutor, forma de manuseio do transdutor) e aquisição correta da imagem para a mensuração do volume vesical^(4,11,19-22)^.

Após a construção do *checklist*, o mesmo foi avaliado por um grupo de dez especialistas na área da USG em relação ao conteúdo, e esta forma de análise das evidências de validade é essencial para fornecer dados sobre o grau em que os elementos de um instrumento de avaliação/*checklist* são relevantes e representativos do construto principal e estão claros quanto à sua ação. A representatividade do grupo de especialistas quanto ao número e à formação profissional é outro fator importante em estudos de validação de conteúdo, e o grupo de especialistas deste estudo apresentava elevada experiência na temática da USG^(28)^.

Para a análise das evidências de validade de conteúdo, foi optado pelo cálculo do IVC, por ser o cálculo estatístico mais utilizado neste tipo de avaliação, e os valores do IVC alcançados neste estudo superaram o ponto de corte estabelecido na literatura de 80%^(16)^. Outro cálculo estatístico adotado neste estudo foi a análise da concordância entre os especialistas, tendo sido utilizado o coeficiente de concordância entre os juízes de segunda ordem de Gwet (AC2 de Gwet), que pode ser utilizado quando há a presença de dois ou mais juízes com uma escala de classificação com duas ou mais categorias, sendo que, quanto mais próximo de 1, menor a probabilidade de a concordância entre os juízes acontecer devido ao acaso. No presente estudo, o coeficiente foi superior ao ponto de corte (0,80) estabelecido^(17)^. A hipótese nula para o AC2 de Gwet, assim como para outras medidas de concordância, é que não há concordância além do que seria esperado ao acaso. O valor elevado de AC2 indica uma concordância significativa entre os avaliadores, sugerindo que a hipótese nula pode ser rejeitada^(29)^.

Dessa forma, a criação de um *checklist* para o estudo vigente se torna essencial para orientar o processo de capacitação dos enfermeiros à beira do leito no que diz respeito à identificação das principais etapas fundamentais para a verificação do volume vesical por meio da USG. Ressalta-se a necessidade de novos estudos que abordem a aplicação desse *checklist* na capacitação dos enfermeiros e sua implicação na precisão desses profissionais na utilização da ferramenta POCUS para a verificação do volume vesical.

### Limitações do estudo

Este estudo desenvolveu um *checklis*t para avaliação do volume vesical pela USG, porém não realizou um pré-teste para sua aplicação nem tampouco sua utilidade no desenvolvimento das competências, sendo identificada esta lacuna como uma limitação do estudo.

### Contribuições para a área da enfermagem

O *checklist* desenvolvido para a avaliação de competências para a realização da USG para mensuração do volume vesical pode ser aplicado em processos educacionais e capacitação dos enfermeiros, pois proporciona uma padronização na avaliação das competências desenvolvidas.

## CONCLUSÕES

O *checklist* desenvolvido por meio de revisão integrativa de literatura para avaliação do desenvolvimento das competências para a mensuração volume vesical por meio da USG foi composto por 23 itens e dividido em três domínios: passos envolvendo a segurança do paciente; manuseio geral dos aparelhos de USG; e aquisição correta da imagem para a mensuração do volume vesical. O *checklist* alcançou adequadas evidências de validade de conteúdo, com IVC superior a 0,80 em todos os itens, e alto nível de concordância entre os avaliadores, podendo ser utilizado na prática educacional nos diversos níveis de formação do enfermeiro.
